# Retrograde Intramedullary Nailing Hardware Failure of a Supracondylar Distal Femur Fracture With Intercondylar Extension

**DOI:** 10.7759/cureus.26276

**Published:** 2022-06-24

**Authors:** Mark D Miller, Jorge Perera, Erin Smith, Jeffrey Burnette

**Affiliations:** 1 Orthopedics, Lake Erie College of Osteopathic Medicine, Sarasota, USA; 2 Orthopedic Surgery, Lake Erie College of Osteopathic Medicine, Bradenton, USA; 3 Orthopedic Surgery, Southeast Orthopedic Specialists, Jacksonville, USA

**Keywords:** distal femoral hardware failure, hardware failure, distal femoral replacement, revision arthroplasty, distal femur fracture

## Abstract

This case report presents the failure of retrograde intramedullary (IM) nailing in a supracondylar distal femur fracture in a 72-year-old female after a fall from standing. Multiple medical comorbidities are a known risk factor for fracture nonunion. With the rising incidence of patients having osteoporosis and multiple medical comorbidities, orthopedic surgeons need to be prepared for the treatment of hardware complications.

The patient is a 72-year-old severely obese female with multiple medical comorbidities including cardiac valvular disease, hypertension, type II diabetes mellitus, hypothyroidism, acute on chronic blood loss anemia, rheumatoid arthritis, and lupus arthritis. She presented after a fall from standing where she sustained a closed displaced left supracondylar distal femur fracture with intercondylar extension.

Open reduction and internal fixation (ORIF) was performed on the left distal femur intercondylar split and retrograde intramedullary nailing for the left supracondylar distal femur fracture. Three-month follow-up X-rays revealed no osseous formation of the supracondylar distal femur fracture and catastrophic failure of the implants with two broken screws and a broken condylar bolt consistent with hardware failure.

Treatment options included either non-weight-bearing for three months to evaluate for callus formation, which would require her to be in a wheelchair, or surgical referral for implant removal and distal femur replacement. The patient elected to undergo revision surgery consisting of distal femoral replacement. Following revision surgery, the patient was discharged with physical therapy referral. She disclosed a decrease in pain and increased range of motion (ROM) compared to the preoperative state.

This case demonstrates an elderly, obese patient with multiple comorbidities including type II diabetes mellitus and autoimmune conditions that placed the patient at high risk for hardware failure following surgery. Due to pain and quality of life concerns, patients with such injuries may be forced into a situation with limited options. This case highlights the need for optimal surgeon-to-patient communication to ensure that patients and all members of their healthcare team are knowledgeable when certain clinical situations are considered high risk for failure. Moving forward, risk factor consideration and medication adjustments are preoperative topics of discussion that should be discussed at length with the patient in order to provide the best opportunity for a successful surgery.

## Introduction

This case report presents the failure of retrograde intramedullary (IM) nailing in a supracondylar distal femur fracture in a 72-year-old female after a fall from standing. The presence of multiple medical comorbidities is a known risk factor for hardware failure. With the rising incidence of patients having osteoporosis and multiple medical comorbidities, orthopedic surgeons need to be prepared to treat hardware failure and the associated hardware complications.

Supracondylar and intercondylar distal femur fractures represent less than 1% of all fractures and only 3%-6% of femur fractures [1]. These rare fractures frequently are the sequelae of high-energy falls or osteoporotic individuals, but the union rate of these traumatic fractures has been reported as high as 97% [2]. When hardware failure does occur following distal femoral fracture repair, this signifies significant challenges to the quality of life for the patient with substantial bone loss and soft tissue scarring [3]. Hardware implementation success is typically assessed at three-month follow-up visits when patients report continued pain at the fracture site or lack of osseous formation on X-rays [3].

The literature contains numerous reports and studies on the generalized treatment of hardware failure of fractures, as well as distal femoral fractures. However, a focused approach to cases such as this has not been extensively reported in the literature. The purpose of this report is to highlight the risk factors that contribute to hardware failure in distal femoral fractures, as well as the importance of patient-to-surgeon communication in instances where a patient’s quality of life can be significantly affected depending on their decisions.

## Case presentation

**Figure 1:**
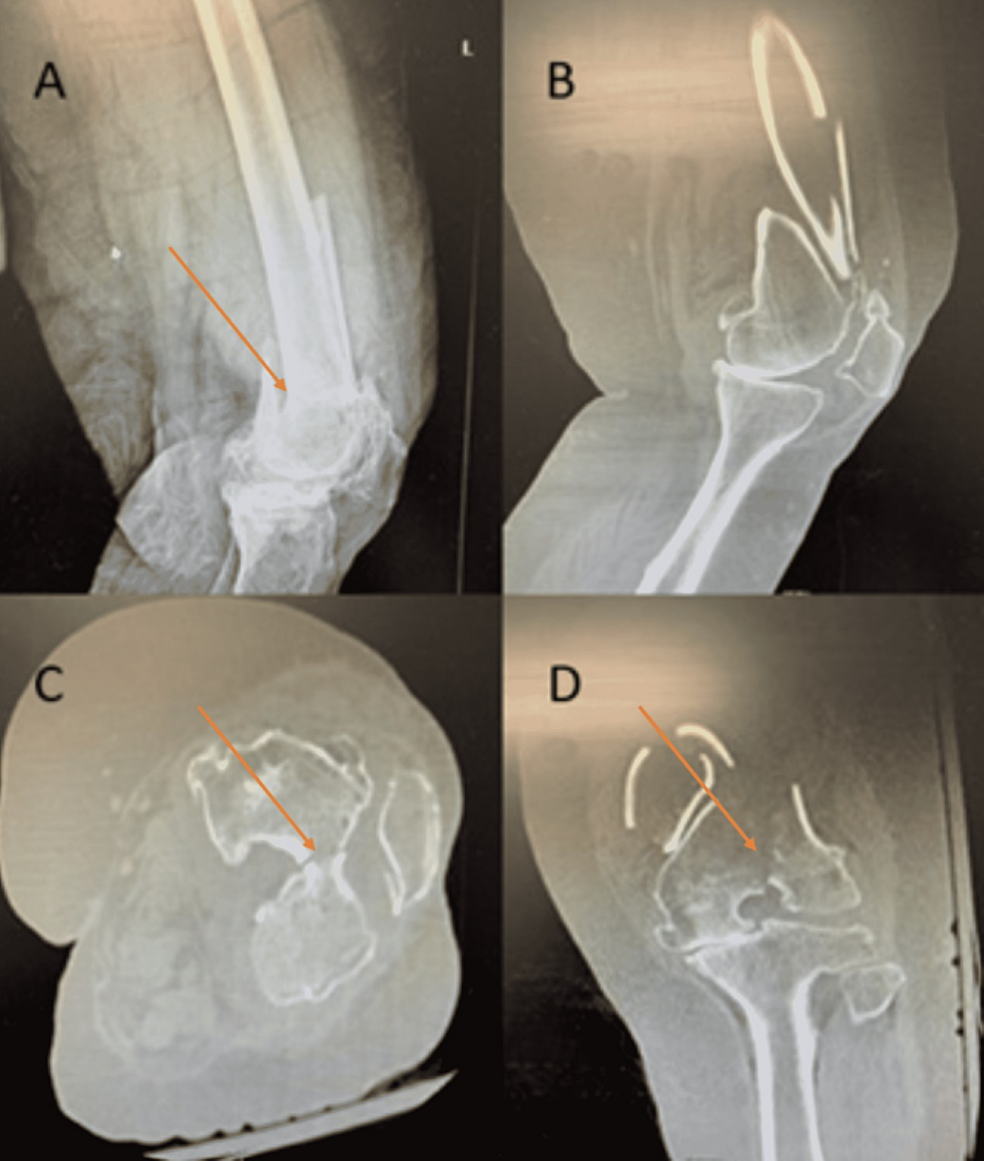
Preoperative imaging revealing OTA class C3 fracture of the left distal femur. A: Lateral X-ray of the left knee. B: Lateral CT of the left knee. C: Axial CT of the left knee. D: Coronal CT of the left knee. Arrows dictate intercondylar extension of the fracture with intact lateral wedge consistent with OTA C2.1 fracture classification. CT: computed tomography

A 72-year-old female was admitted to trauma surgery service after a fall from standing, where she sustained a closed displaced left supracondylar distal femur fracture with intercondylar extension with an intact lateral wedge (Orthopedic Trauma Association (OTA) classification C2.1) (Figure 1) [1]. Her past medical comorbidities were extensive, including cardiac valvular disease, hypertension, type II diabetes mellitus, hypothyroidism, acute on chronic blood loss anemia, rheumatoid arthritis, and lupus arthritis, which were treated with anticoagulation and immunosuppressant medications.

**Figure 2:**
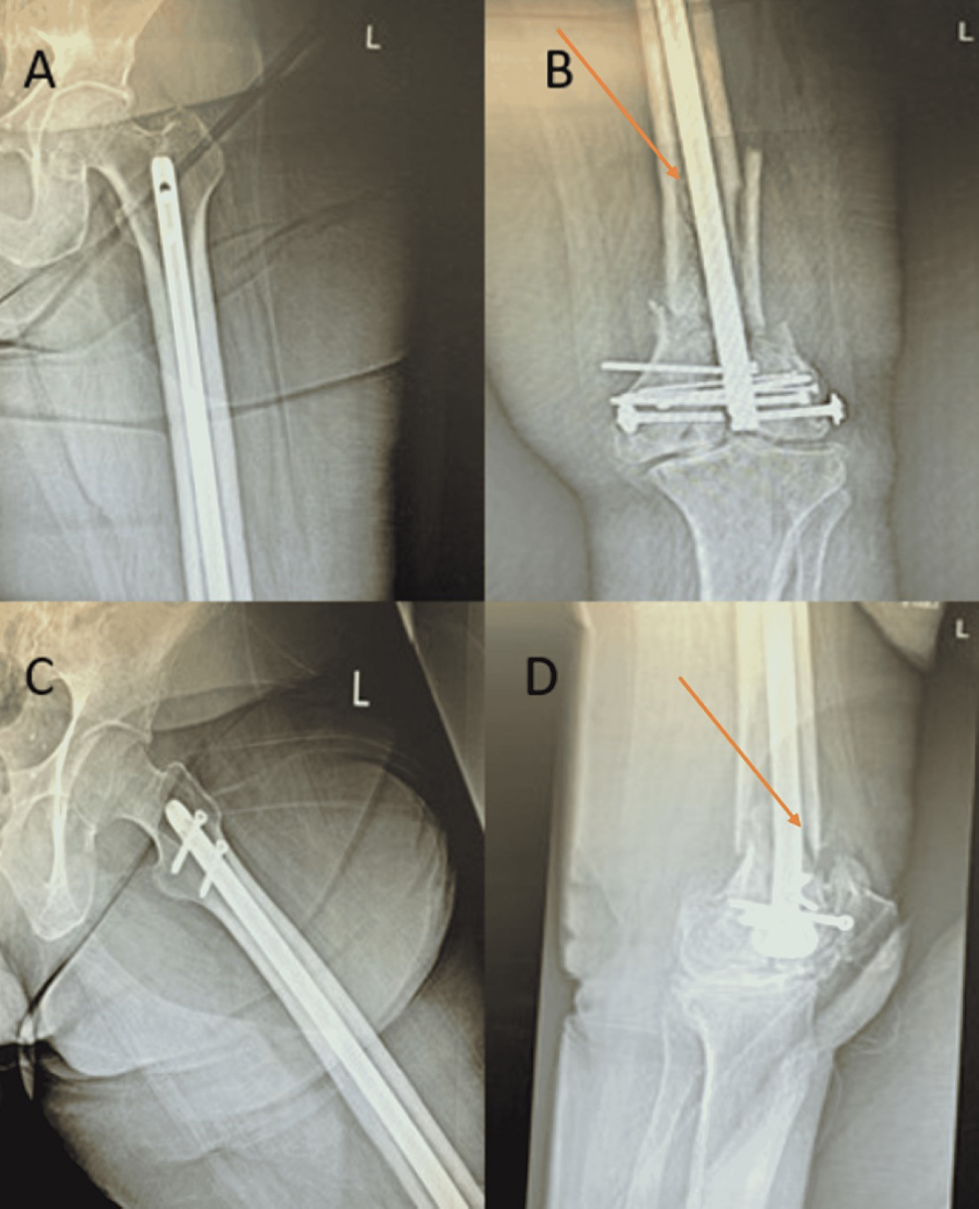
Three-month postoperative imaging revealing hardware failure of distal femur fracture. A: AP X-ray of the left hip. B: AP X-ray of the left knee. C: Frog-leg X-ray of the left hip. D: Lateral X-ray of the left knee. Arrows indicate a lack of osseous formation.

The patient underwent operative reduction and internal fixation (ORIF) of the left distal femur with retrograde intramedullary (IM) nailing for the left supracondylar distal femur fracture. An 11 × 380 mm supracondylar nail was used and impacted into place to the appropriate depth. Five interlocking screws, an intercondylar bolt, two 3.5-mm lag screws, and a set screw were used and placed. The postoperative plan included weight-bearing as tolerated in the left lower extremity and range of motion (ROM) as tolerated in the left hip and knee. Deep venous thrombosis (DVT) prophylaxis was given, as well as calcium, vitamin D, and ergocalciferol protocol. Three-month follow-up X-rays showed no osseous formation of the supracondylar distal femur fracture and catastrophic failure of the implants with two broken screws and a broken condylar bolt consistent with hardware failure (Figure 2).

**Figure 3:**
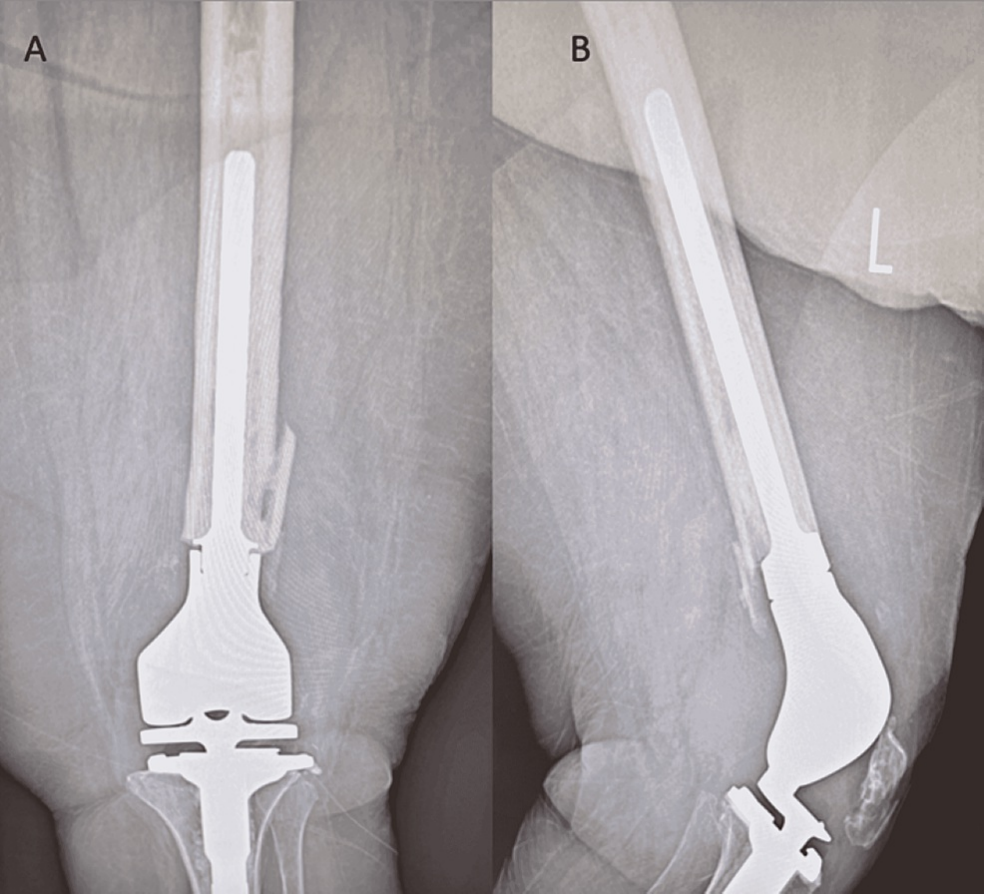
Post-revision surgery imaging demonstrating distal femoral replacement. A: AP X-ray view of the left knee. B: Lateral view X-ray of the left knee revealing patella infera.

Treatment options included either non-weight-bearing for three months to evaluate for callus formation, which would require her to be in a wheelchair, or surgical referral for implant removal and distal femur replacement. The patient elected to undergo revision surgery. Revision surgery consisted of hardware extraction followed by distal femoral replacement, utilizing cemented implants. Following revision surgery, the patient was able to be discharged home with physical therapy. Her pain and range of motion have improved dramatically since revision surgery, with a six-week follow-up revealing an improvement in flexion from 20 degrees to 90 degrees (Figure 3).

## Discussion

Supracondylar and intercondylar femur fractures are severe injuries that frequently present with complications. These femur fractures may represent less than 1% of all fractures and only 3%-6% of femur fractures, but due to the aging population and the rising prevalence of medical comorbidities, these fractures are likely to see an increase in prevalence [1]. Distal femur fractures tend to be bimodally distributed, with the vast majority of patients either being young adults involved in high-velocity accidents or elderly osteoporotic individuals who experience a fall from standing [1].

The OTA fracture classification system is the most widely utilized method for specific fracture diagnosis [1]. The fractures are classified as type A (extra-articular), type B (partial articular involvement/unicondylar), and type C (complete articular involvement/bicondylar) [1]. Subclassification within the fracture types A and C indicates the degree of comminution (A2/A3, as well as C2, involve metaphyseal comminution). Type C3 fracture reflects metaphyseal and intercondylar comminution. Type B fractures are subclassified based on the involved femoral condyle, with B1 and B2 reflecting the lateral and medial condyles, respectively. The fracture designation of B3 refers to the “Hoffa” coronal partial articular fracture [1]. This case presentation refers to an OTA class C2.1 fracture, which is typically treated with either ORIF and/or retrograde IM nailing, with ORIF reporting lower rates of hardware failure [4]. Following hardware failure, the distal femoral replacement has been cited as a valuable treatment in elderly osteoporotic individuals with a low likelihood of adequate bone growth potential [5].

Hardware failure, albeit a difficult term to define, can be considered the failure of a construct to provide adequate stability and fixation for fracture union. The risk factors for hardware failure is a highly debated and well-researched topic by orthopedic surgeons. Aside from the type of fracture, age of the patient, and lifestyle habits (i.e., smoking), the patient, in this case, had multiple comorbidities that may have led to the hardware failure. With the low-impact (fall from height) fracture of the femur, the patient can be accurately diagnosed with osteoporosis [6]. Severe osteoarthritis in this patient placed severe stress on the fixation within the supracondylar region, and this extensive stress placed upon the interlocking screws likely led to the hardware failure.

It has been reported that distal femoral fracture hardware failure rates can be as high as 20%, with the majority of implant failures occurring within the first year after hardware implantation (80%) [7]. Distal femoral locked plating has been described as the standard of care for distal femoral fractures and has been reported with lower rates of hardware failure than IM nailing alone [8]. Given this patient’s osteoporotic state and the comminuted state of her fracture, initial treatment with a distal femoral replacement would have been the optimal option to reduce her risk of hardware failure. Distal femoral replacement for comminuted distal femoral fractures in the elderly population has been shown to produce fewer hardware failure rates, being reported at 7%-10% [9]. Further research is needed to elucidate the mechanisms by which these risk factors may contribute to hardware failure.

## Conclusions

With the risk of hardware failure being a subject with sufficient research, the question is for the surgeon to consider the best next step on a case-by-case basis in patients with high-risk femoral fractures. In this specific case, an elderly, obese patient with a lengthy medication list and multiple comorbidities such as diabetes and autoimmune conditions warrants a lengthy discussion on the risks versus rewards of the surgery, as well as the rehabilitation period following. Due to pain and quality of life concerns, patients with such injuries may be forced into a situation with limited options. Surgeons need to ensure that they and the patients are knowledgeable when certain clinical situations are considered high risk for failure. Moving forward, risk factor consideration and medication adjustments are preoperative topics of discussion that need to be openly discussed with the patent in order to provide the best opportunity for a successful surgery.
